# Thermal Conductive 2D Boron Nitride for High‐Performance All‐Solid‐State Lithium–Sulfur Batteries

**DOI:** 10.1002/advs.202001303

**Published:** 2020-08-20

**Authors:** Xuesong Yin, Liu Wang, Yeongae Kim, Ning Ding, Junhua Kong, Dorsasadat Safanama, Yun Zheng, Jianwei Xu, Durga Venkata Maheswar Repaka, Kedar Hippalgaonkar, Seok Woo Lee, Stefan Adams, Guangyuan Wesley Zheng

**Affiliations:** ^1^ Institute of Materials Research and Engineering A*STAR (Agency for Science, Technology and Research) Singapore 138634 Singapore; ^2^ Department of Chemical and Biomolecular Engineering National University of Singapore Singapore 117585 Singapore; ^3^ School of Electrical and Electronic Engineering Nanyang Technological University Singapore 639798 Singapore; ^4^ Department of Materials Science and Engineering National University of Singapore Singapore 117576 Singapore

**Keywords:** all‐solid‐state lithium–sulfur batteries, boron nitride, solid polymer electrolytes, uniform lithium deposition

## Abstract

Polymer‐based solid‐state electrolytes are shown to be highly promising for realizing low‐cost, high‐capacity, and safe Li batteries. One major challenge for polymer solid‐state batteries is the relatively high operating temperature (60–80 °C), which means operating such batteries will require significant ramp up time due to heating. On the other hand, as polymer electrolytes are poor thermal conductors, thermal variation across the polymer electrolyte can lead to nonuniformity in ionic conductivity. This can be highly detrimental to lithium deposition and may result in dendrite formation. Here, a polyethylene oxide‐based electrolyte with improved thermal responses is developed by incorporating 2D boron nitride (BN) nanoflakes. The results show that the BN additive also enhances ionic and mechanical properties of the electrolyte. More uniform Li stripping/deposition and reversible cathode reactions are achieved, which in turn enable all‐solid‐state lithium–sulfur cells with superior performances.

## Graphical abstract

A boron nitride (BN) incorporated solid polymer electrolyte is developed with improved ionic conductivity, thermal responses, and mechanical properties for the application of all‐solid‐state Li–S batteries. The thermal and current uniformity derived from BN additive leads to a uniform Li deposition and preferred S conversion. Thus, an all‐solid‐state Li–S battery is successfully demonstrated with outstanding electrochemical performances.

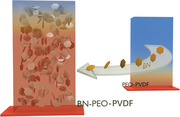

All‐solid‐state batteries with high capacity electrode materials provide a promising route for the next‐generation batteries that combine high energy density and advanced safety features.^[^ ^1^, ^2^, ^3^, ^4^, ^5^, ^6^ ^]^ Solid‐state electrolytes with high ionic conductivity and electronic insulation are considered as the key component of all‐solid‐state batteries to replace flammable liquid electrolyte and separator. Polyethylene oxide (PEO)‐based electrolytes have been widely explored in solid‐state lithium batteries due to their reasonably good ionic conductivity at elevated temperature, decent interfacial characteristics with different electrode materials, and good compatibility with scaled‐up production process.^[^ ^7^, ^8^, ^9^, ^10^, ^11^, ^12^ ^]^ One common drawback of PEO‐based electrolytes is the requirement to operate at elevated temperature, due to their limited ionic conductivity at room temperature (RT).^[^ ^13^ ^]^ Considerable efforts have been made to address this issue through approaches such as copolymerization, polymer blending, incorporation of inorganic fillers (both Li‐ion conductors and insulators).^[^ ^7^, ^14^, ^15^, ^16^, ^17^ ^]^ Moreover, since the ionic conductivity of PEO‐based electrolytes is highly temperature‐dependent,^[^ ^18^, ^19^ ^]^ thermal variation across the electrolyte can lead to nonuniform reaction in the electrode. This can significantly affect lithium deposition and lead to dendrite formation.^[^ ^20^ ^]^ Therefore, improving the thermal uniformity of polymer electrolyte is critical for the performance of high capacity all‐solid‐state batteries. However, such thermal character of PEO‐based electrolyte is not well explored.^[^ ^21^ ^]^ Here we use 2D boron nitride (BN) to improve the thermal uniformity of PEO‐based polymer electrolyte and demonstrate its outstanding performance in all‐solid‐state lithium–sulfur (Li–S) batteries. The results show the importance of rapid thermal activation in improving the uniformity of lithium reaction and cycling stability.

As illustrated in **Figure** **1**a, polymer electrolyte is not a good thermal conductor and the heat transport in electrolyte becomes the limiting factor which could inhibit thermal responses of such solid‐state Li batteries.^[^ ^22^ ^]^ Boron nitride has been recognized as a good thermal conductor and effective additive to modify the thermal and mechanical properties of polymer composites.^[^ ^23^, ^24^, ^25^, ^26^, ^27^ ^]^ In addition, its low electrical conductivity and good electrochemical stability also mean that the additive will not participate in the battery reactions and can remain stable over long‐term cycling.^[^ ^28^, ^29^ ^]^ BN was reported to advance Li batteries with liquid‐based electrolytes,^[^ ^30^, ^31^, ^32^, ^33^ ^]^ and serve as effective interfacial stabilizer against Li anode or filler in polymer electrolytes for Li‐ion batteries because of its electrochemical inertness and mechanical robustness.^[^ ^29^, ^34^, ^35^ ^]^ However, the impact of its thermal property on the solid‐state Li metal battery with high‐capacity S cathode has not been well examined. In this work, 2D BN nanoflakes are introduced into a blended PEO‐PVDF (poly(vinylidene fluoride))‐LiTFSI (lithium bis(trifluoromethanesulfonyl)imide) polymer to form a novel solid‐state composite electrolyte (see Figure 1b). This electrolyte presents unique properties combining high thermal conductivity, electrical insulation, and electrochemical stability. The BN additive is found to possess multiple functions in terms of increasing ionic conductivity, mechanical strength, and heat transport of the PEO‐based electrolyte. As a result, high‐performance all solid‐state Li–S batteries are achieved with high specific capacity, good cycling stability, and rate capability, owing to the uniform and compact Li deposition and favorable S conversion during charge–discharge cycles.

**Figure 1:**
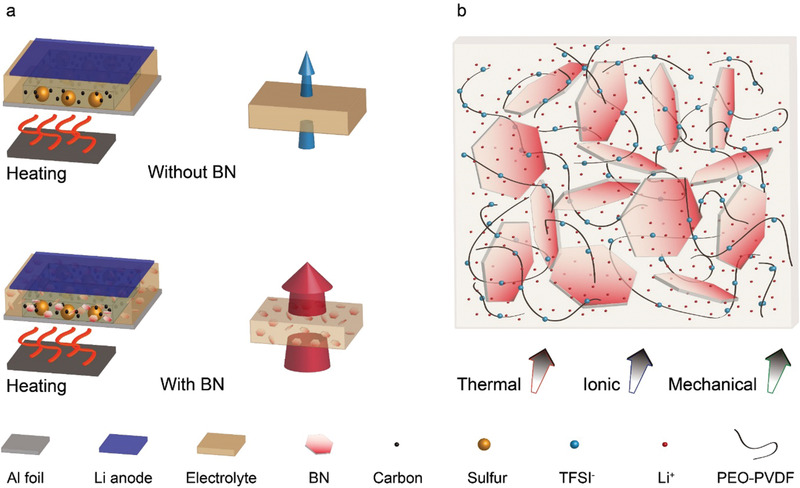
Rational design of solid‐state polymer electrolyte with BN additive. a) Illustration of solid‐state Li–S batteries with polymer‐based electrolytes and schematic comparison of heat transport through electrolytes with and without BN additives and b) sketch of a composite electrolyte consisting of 2D BN flakes and a blended PEO‐PVDF polymer with LiTFSI.

Free‐standing PEO‐PVDF and BN‐PEO‐PVDF electrolyte membranes are prepared through a tape‐casting process followed by delamination as described in the Experimental Section. Scanning electron microscopy (SEM) and optical images of the PEO‐PVDF and BN‐PEO‐PVDF electrolytes are shown in **Figure** **2**a,b. Compared with the translucent PEO‐PVDF sample, the white BN‐PEO‐PVDF sample has smaller grain textures and traces of BN nanoparticles on its surface. Additional information of the structures of BN nanoflakes and electrolytes are provided in Figure S1 in the Supporting Information. As seen from Figure 2c, the ionic conductivities of both PEO‐PVDF and BN‐PEO‐PVDF electrolytes increase when the temperature increases. At an elevated temperature (>60 °C), the ionic conductivity of the BN‐PEO‐PVDF sample (≈10^−4^ S cm^−1^) is higher than the PEO‐PVDF sample (≈10^−5^ S cm^−1^). Meanwhile, as revealed by the differential scanning calorimetry (DSC) measurements (Figure 2d), BN‐PEO‐PVDF has lower glass transition (*T*_g_) and melting (*T*_m_) temperatures than PEO‐PVDF. It suggests the BN additive interferes with PEO‐based electrolyte, increases structural disorders, and improves its ionic conductivity.^[^ ^7^ ^]^ In Figure 2d, the load (*P*)–displacement (*h*) curves demonstrate a larger modulus of the BN‐PEO‐PVDF sample than the PEO‐PVDF one. The enhanced mechanical strength is believed to help suppress the formation of porous Li metal structures during battery operation.^[^ ^8^ ^]^ To verify this point, Li^0^ symmetric cells with PEO‐PVDF and BN‐PEO‐PVDF electrolytes are tested at 70 °C in Figure 2f. The overpotential of the BN‐PEO‐PVDF cell when cycled at a current density of 0.1 mA cm^−2^ retains a small value (≈40 mV) for more than 200 h, while the PEO‐PVDF cell exhibits a gradually increased overpotential to about 100 mV followed by a sign of short circuit after only 55 h. The lower overpotential and longer cycling time of the BN‐PEO‐PVDF cell imply more effective and stable Li metal stripping and deposition during cycling. Instead of porous Li metal structures formed in the PEO‐PVDF cell, dense and flattened Li metal deposition is revealed in the cell with BN‐PEO‐PVDF electrolyte after cycling (Figure 2g). Additionally, the BN‐PEO‐PVDF and PEO‐PVDF electrolytes are of the same Li‐ion transfer number as characterized in Figure S2 and Table S1 in the Supporting Information. The thermogravimetric analysis (TGA) and linear sweep voltammetry tests curves in Figure S3 in the Supporting Information suggest good thermal and anodic stabilities of PEO‐PVDF and BN‐PEO‐PVDF electrolytes as well. Overall, BN incorporation into the PEO‐PVDF blend electrolyte results in favorable properties of high ionic conductivity and mechanical strength together with good thermal and electrochemical stabilities for Li battery applications.

**Figure 2:**
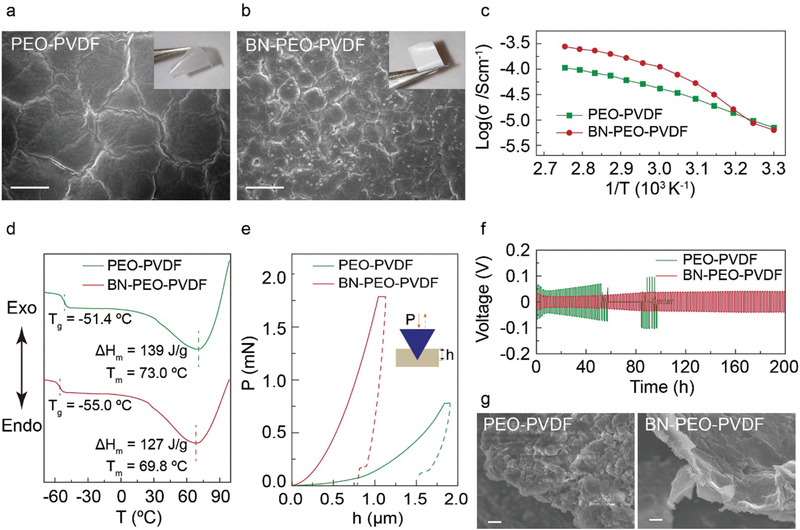
Characterizations of BN‐PEO‐PVDF and PEO‐PVDF electrolytes. SEM and optical images of a) PEO‐PVDF and b) BN‐PEO‐PVDF electrolyte membranes. c) Change of ionic conductivity with respect to temperature (log*σ* vs 1/*T*), d) DSC profiles, e) loading force (*P*) as a function of indentation displacement (*h*) during loading and unloading tests for PEO‐PVDF and BN‐PEO‐PVDF electrolytes. f) Galvanostatic cycling of Li^0^ symmetric cells with PEO‐PVDF and BN‐PEO‐PVDF electrolytes at a current density of 0.1 mA cm^−2^ and g) SEM images of deposited Li metal in the symmetric cells after cycling. Scale bar: 10 µm in (a) and (b) and 1 µm in (g).

More interestingly, the BN additive is found to have significant effects on the thermal responses of PEO‐based electrolytes. Thermal diffusivity (*α*) and heat capacity (*c*) of the PEO‐PVDF and BN‐PEO‐PVDF samples are measured at the operation temperature (70 °C) in **Figure** **3**a. Thermal conductivity (*κ*) is then calculated as *κ* = *α* · *c* · *ρ*, where *ρ* is density. It is found that the BN‐PEO‐PVDF electrolyte has considerably higher *α* and *κ* values than the PEO‐PVDF one, which is attributed to the high thermal conductive nature of BN.^[^ ^36^ ^]^ The thermal and electrical parameters of the electrolytes and BN are shown in Table S2 in the Supporting Information. We carried out thermal testing on PEO‐PVDF and BN‐PEO‐PVDF films and their temperature changes were recorded by infrared (IR) images in Figure 3b. A distinctive temperature difference between PEO‐PVDF and BN‐PEO‐PVDF samples is observed after heating for 5 s. It implies an accelerated thermal transport when BN flakes are added into the PEO‐PVDF electrolyte. Accordingly, the ionic conductivity of BN‐PEO‐PVDF electrolyte should also present a faster relaxation upon a temperature change. As demonstrated in Figure 3c, when the BN‐PEO‐PVDF and PEO‐PVDF electrolytes are subjected to a temperature switch from RT to 70 °C, the ionic conductivity of BN‐PEO‐PVDF ramps up to a stable value of about 2 × 10^−4^ S cm^−1^ within 300 s, but the PEO‐PVDF sample takes more than 700 s to reach a smaller stabilized value of about 0.6 × 10^−4^ S cm^−1^. Because of the higher ionic conductivity of BN‐PEO‐PVDF electrolyte, the Li–S cell with the BN‐PEO‐PVDF electrolyte holds a relatively higher discharge potential than the cell with PEO‐PVDF electrolyte at all the testing temperatures in Figure S4 in the Supporting Information. Meanwhile, the faster thermal response of the BN‐PEO‐PVDF electrolyte also results in a quicker stabilization of discharge potentials in the BN‐PEO‐PVDF based Li–S cell when temperature changes (see Figure S4, Supporting Information).

**Figure 3:**
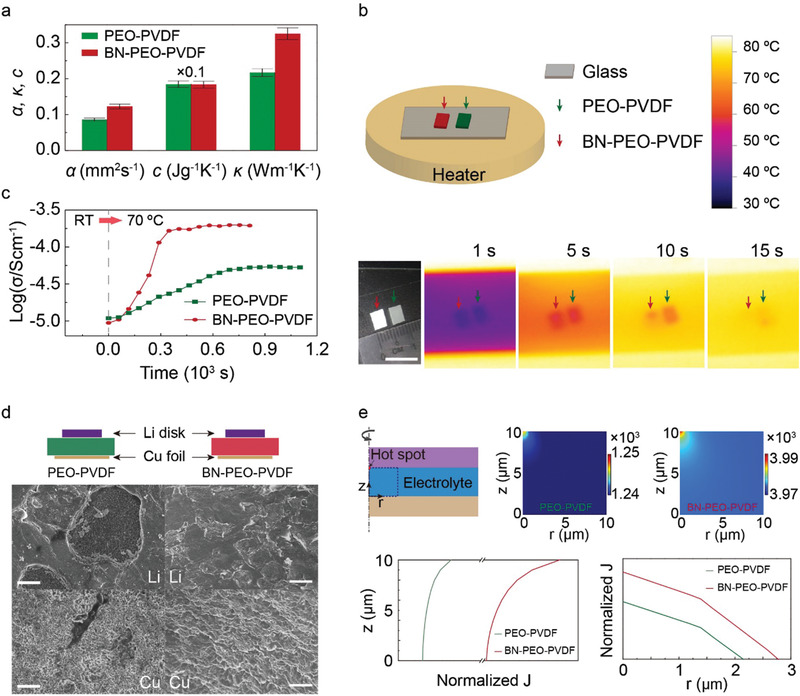
Thermal responses of PEO‐PVDF and BN‐PEO‐PVDF electrolytes. a) Thermal diffusivity (*α*), heat capacity (*c*), and thermal conductivity (*κ*) of PEO‐PVDF and BN‐PEO‐PVDF electrolytes. b) Temperature changes of PEO‐PVDF and BN‐PEO‐PVDF films with respect to heating time characterized by infrared images. c) Changes of ionic conductivities of PEO‐PVDF and BN‐PEO‐PVDF upon a temperature change from RT to 70 °C. d) Sketch of Li–Cu cells with PEO‐PVDF and BN‐PEO‐PVDF electrolyte and SEM images of Li metal on the surfaces of Li disks and Cu foils after 2 mAh cm^−2^ Li deposition at 1 mA cm^−2^. e) Simulated current density (*J*) changes due to a hot spot in PEO‐PVDF and BN‐PEO‐PVDF electrolyte: a sketch of the model, variations of *J* near the heat source (squared area in the model) at *t* = 0.5 s (see Figure S5 and Video S1, Supporting Information) and normalized *J* distribution along directions normal (*z*) and parallel (*r*) to the electrode surface (see Figure S6, Supporting Information). Scale bar: 1 cm in (b), 10 µm in (d).

Electrochemical behaviors of Li anode with PEO‐PVDF and BN‐PEO‐PVDF electrolytes are characterized in Li–Cu asymmetric cells in Figure 3d. Li metal with a capacity of 2 mAh cm^−2^ was plated on Cu foil at a current density of 1 mA cm^−2^. After Li deposition, significant pits are observed on the surface of Li foil in the cell with PEO‐PVDF electrolyte, and correspondingly the deposited Li on the Cu side exhibits unevenly distributed dense and porous regions (PEO‐PVDF column in Figure 3d). However, in the cell with BN‐PEO‐PVDF electrolyte, a shallower and more uniform stripping pattern is observed on the Li surface. The plated Li metal on the Cu foil is also more uniform (BN‐PEO‐PVDF column in Figure 3d). In Figure 3e, the variations of current density (*J*) resulted from a hot spot in the PEO‐PVDF and BN‐PEO‐PVDF electrolytes were simulated. The cross‐sectional current density mappings at 0.5 s show that the BN‐PEO‐PVDF electrolyte show better uniformity in current density than the PEO‐PVDF sample (Figure S5 and Video S1, Supporting Information). The plots of normalized current density along the directions normal (*z*) and parallel (*r*) to the electrode surface (Figure S6, Supporting Information) also indicate a wider distribution of current densities in the BN‐PEO‐PVDF electrolyte in addition to its higher current density value. More details about the simulation can be found in the Experimental Section and Figures S5 and S6 and Video S1 in the Supporting Information. The results suggest that BN‐PEO‐PVDF electrolyte can help reduce the temperature‐induced variation in current density more effectively, so more uniform Li stripping and deposition in the BN‐PEO‐PVDF cell in Figure 3d are observed.

Battery performances of solid‐state Li‐S cells with BN‐PEO‐PVDF and PEO‐PVDF electrolytes are evaluated at 70 °C in **Figure** **4** and Figures S6 and S7 in the Supporting Information. In Figure S7 in the Supporting Information, cyclic voltammetry (CV) and charge–discharge curves of cathode without S do not show any capacity, which confirm the electrochemical inertness of BN within the operational potential range of a Li–S cell. The charge–discharge profiles in Figure 4a,b show a typical curve for Li–S chemistry with two dominant discharge plateaus at around 2.4 and 2.0 V. However, the cell with PEO‐PVDF electrolyte has a relatively large overpotential and shortened voltage plateau than the BN‐PEO‐PVDF cell, which suggests that the BN additive reduces the cell impedance and improves the reaction kinetics in the solid‐state Li–S cell. Moreover, the SEM images and energy‐dispersive X‐ray (EDX) mappings of element S in the cathodes of BN‐PEO‐PVDF and PEO‐PVDF cells before and after cycling are shown in Figure 4c,f. In contrast to notable structural defects and unevenly distributed S signals detected in the PEO‐PVDF cell after cycling (Figure 4f), the cathode of BN‐PEO‐PVDF cell remains intact with uniform S distributions in Figure 4c. The uniform temperature distribution in the electrolyte with BN incorporation helps achieve uniform current density and hence homogeneous conversion reactions in the cathode. Therefore, sulfur remains evenly distributed in the cathode with BN after cycling (Figure 4c). In contrast, the sulfur conversion is more localized in the cell without BN. The nonuniform volume changes at certain area in the cathode lead to voids or cracks after cycling (Figure 4f). Because of these beneficial effects of BN additive on both Li anode and S cathode during electrochemical reactions, the BN‐PEO‐PVDF cell possesses much improved battery performances. The all‐solid‐state Li–S battery with BN‐PEO‐PVDF electrolyte successfully delivers higher reversible specific capacities of ≈1200 mAh g^−1^‐S during initial cycles at 1/20 C and ≈790 mAh g^−1^‐S after 50 cycles at 1/10 C when compared to the cell with PEO‐PVDF electrolyte in Figure 4d and Figure S8b in the Supporting Information. A coulombic efficiency above 95% is achieved after the initial four cycles, indicating the good reversibility of the electrochemical reactions in the cell with BN‐PEO‐PVDF electrolyte (Figure S8c, Supporting Information). Moreover, a higher rate performance is observed in the BN‐PEO‐PVDF cell in Figure 4e, which presents high capacities of 750 and 400 mAh g^−1^‐S at 1/5 C and 1/2 C, respectively. As a comparison, the PEO‐PVDF cell only provides 300 mAh g^−1^‐S at 1/5 C and a negligible capacity at 1/2 C. Additionally, battery performance was evaluated at a lower temperature of 55 °C as shown in Figure S9 in the Supporting Information. The BN‐PEO‐PVDF cell gives ≈1000 mAh g^−1^‐S of discharge capacity at 1/20 C and ≈700 mAh g^−1^‐S at 1/10 C with a characteristic two‐plateau feature. While the PEO‐PVDF cell only shows ≈670 and ≈100 mAh g^−1^‐S at 1/20 and 1/10 C, respectively. The better behaviors of BN‐PEO‐PVDF cells than PEO‐PVDF cells at 70 and 55 °C verify that the effectively improved thermal responses of the BN incorporated polymer electrolyte help advance performances of solid‐state Li–S battery.

**Figure 4:**
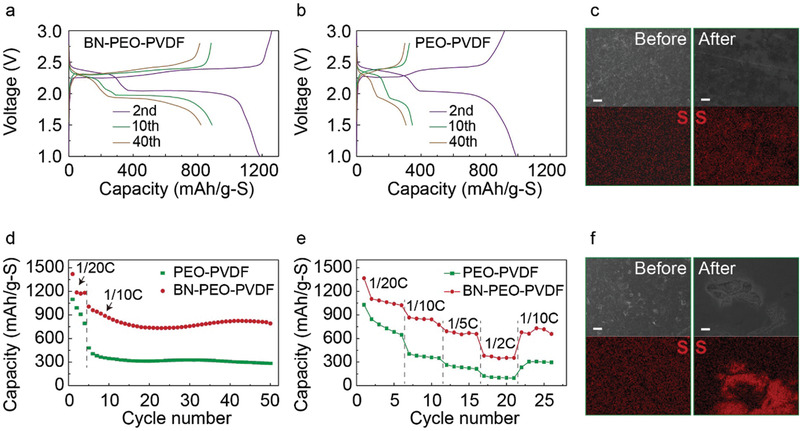
All‐solid‐state Li–S battery performances at 70 °C with PEO‐PVDF and BN‐PEO‐PVDF electrolytes. Charge–discharge curves of solid‐state Li–S cells with a) BN‐PEO‐PVDF and b) PEO‐PVDF electrolyte at various cycles, SEM images of the cathodes in the Li–S cells with c) BN‐PEO‐PVDF and f) PEO‐PVDF electrolyte and their corresponding EDX elemental mapping of S before and after cycling, d) cycling stability and e) rate performance of the Li–S cells with BN‐PEO‐PVDF and PEO‐PVDF electrolytes. Scale bar: 10 µm in (c) and (f).

A PEO‐PVDF blended polymer electrolyte with 2D BN nanoflakes (BN‐PEO‐PVDF) is developed for all‐solid‐state Li–S battery. In addition to the improved ionic and mechanical properties, the BN additive also promotes the thermal responses of PEO‐based electrolyte. It enables a faster equalization of heat variation and more homogenous ionic transport in the BN‐PEO‐PVDF electrolyte. As a result, both Li metal anode and S cathode exhibit more uniform and stable transformations during electrochemical reactions in the Li–S cell with BN‐PEO‐PVDF electrolyte. Outstanding battery performances in terms of high specific capacity, good cyclic and rate behaviors are also achieved in the cells using BN‐PEO‐PVDF electrolyte. This work provides an effective way to modify the thermal characteristics of polymer electrolytes and elaborates its significant influences on the microscopic electrochemical reactions and device performances of all‐solid‐state Li–S batteries.

## Experimental Section

##### Solid Electrolyte Membrane Preparation

Poly(ethylene oxide) (PEO, *M*_v_ 100 000, Aldrich), PVDF (SOLEF 5130), and LiTFSI (Aldrich, 99.95%) with a ratio of 3:1:1 were preweighted in an Ar‐filled glovebox (MBRAUN, Labmaster 200). The powder mixture was dissolved in 1‐methyl‐2‐pyrrolidone (NMP, Aldrich, 99.5%) with constant stirring for 12 h. Then, the slurry was casted on an Al foil and dried at 80 °C for 5 h. Free‐standing PEO‐PVDF electrolyte membrane with a thickness around 20–30 µm was peeled off from the substrate. BN‐incorporated PEO‐PVDF electrolyte (BN‐PEO‐PVDF) was produced through the same procedure, but 12 wt% BN particles (Sigma‐Aldrich, 98%) was added when mixing PEO, PVDF, and LiTFSI powders.

##### Composite S Cathode Fabrication

Elemental S (Alfa Aesar, 99.5%) and conductive carbon (Ketjen black, EC‐600J) were ball milled for 24 h to form a S/C powder with a S:C ratio of 9:1. The cathode slurry was prepared by mixing the S/C powder, PEO‐PVDF (or BN‐PEO‐PVDF) electrolyte, and additional conductive carbon in NMP with constant stirring for 12 h. The slurry was then casted on a carbon‐coated Al foil. The composite S cathodes, consisting of 40 wt% S, 15 wt% conductive carbon, and 45 wt% PEO‐PVDF/BN‐PEO‐PVDF electrolytes, were obtained after drying for 5 h at 80 °C. The mass loading of active material (S) is controlled around 0.8–1.0 mg cm^−2^.

##### Cell Assembling

The composite S cathodes and PEO‐PVDF /BN‐PEO‐PVDF electrolyte membranes were dried and transferred into an Ar‐filled glovebox. The S cathodes, electrolyte membranes, and Li metal foils (China Energy Lithium, 99.9%) were stacked in a 2032 coin‐cell case together with a stainless‐steel spacer and a coned‐disc spring. The all‐solid‐state Li–S cell was sealed by a hydraulic crimping machine (MSK 110).

##### Materials Characterization

The morphologies of the electrolyte, Li metal, and cathode were characterized by a field‐emission scanning electron microscopy (FESEM) (JEOL, JSM 7600F). The elemental distribution was measured by an EDX analyzer attached in the FESEM system. The crystalline structures of the electrolytes were analyzed by X‐ray diffraction (Bruker 2D Micro XRD). Thermal diffusivity, DSC, and heat capacity of the electrolytes were measured by a laser flash apparatus (Netzsch LFA457) and a differential scanning calorimetry system (Mettler Toledo, DSC1), respectively. Thermogravimetric analysis was carried out using a TGA system (TA Q600/2960). Mechanical properties of the electrolytes were characterized by an indentation system (MTS Nano Indenters XP, TN, USA). IR images were captured by a thermal imaging camera (Testo 865).

##### Electrochemical Characterization

The CV and charge–discharge curves were measured at 70 °C by battery testers (Novonix HPC and Arbin). Electrochemical impedance spectroscopy measurements were conducted using an electrochemical workstation (Biologic SP‐300). The AC voltage perturbation amplitude was 10 mV and the frequency range was from 3M Hz to 100 Hz. The testing cells were all connected in heating chambers with temperature controllers.

##### Simulation Analysis

COMSOL simulation was conducted to compare the ionic diffusion in the different solid electrolytes of PEO‐PVDF and BN‐PEO‐PVDF using a finite element package (COMSOL Multiphysics, COMSOL Inc.). For the simulation (Figure S5, Supporting Information), a model consisting of solid electrolyte between Li and Cu foil was constructed. The initiation of Li deposition was depicted as a hot spot at the top surface of solid electrolyte. Material properties of solid electrolytes such as ionic conductivity, thermal conductivity, heat capacity, and density were obtained from experiments. To calculate the current density in the electrolytes, DC current and heat transfer modules were used in order to reflect material properties depending on the temperatures and the results were shown through time‐dependent model studies. The time range was set from 0 to 1 s, and the 1 s means when the temperature generated at the hot spot was saturated in the electrolytes. The current density distributions near the electrode/electrolyte interfaces were also discussed (Figure 3e and Figure S6, Supporting Information).

## Conflict of Interest

The authors declare no conflict of interest.

## Supporting information

Supporting Information

Video S1
