# Supplementary material for: Thermal Conductive 2D Boron Nitride for High‐Performance All‐Solid‐State Lithium–Sulfur Batteries
Source: Adv Sci (Weinh). 2020 Aug 20;7(19):2001303. doi: 10.1002/advs.202001303 (PMC7539184; doi:10.1002/advs.202001303)
Supplement: Supplementary file 1 — Supporting Information [file ADVS-7-2001303-s001.pdf]

© 2020 Wiley-VCH GmbH

ADVANCED SCIENCE
Open Access

Supporting Information

for *Adv. Sci.,* DOI: 10.1002/advs.202001303

Thermal Conductive 2D Boron Nitride for HighPerformance All-Solid-State Lithium–Sulfur Batteries

*Xuesong Yin, Liu Wang, Yeongae Kim, Ning Ding, Junhua Kong, Dorsasadat Safanama, Yun Zheng, Jianwei Xu, Durga Venkata Maheswar Repaka, Kedar Hippalgaonkar, Seok Woo Lee, Stefan Adams, and Guangyuan Wesley Zheng**

Copyright WILEY-VCH Verlag GmbH & Co. KGaA, 69469 Weinheim, Germany, 2018.

Supporting Information

Thermal conductive two-dimensional boron nitride for high-performance all-solid-state lithium-sulfur batteries

*Xuesong Yin, Liu Wang, Yeongae Kim, Ning Ding, Junhua Kong, Dorsasadat Safanama, Yun Zheng, Jianwei Xu, D. V. Maheswar Repaka, Kedar Hippalgaonkar, Seok Woo Lee, Stefan Adams, Guangyuan Wesley Zheng**

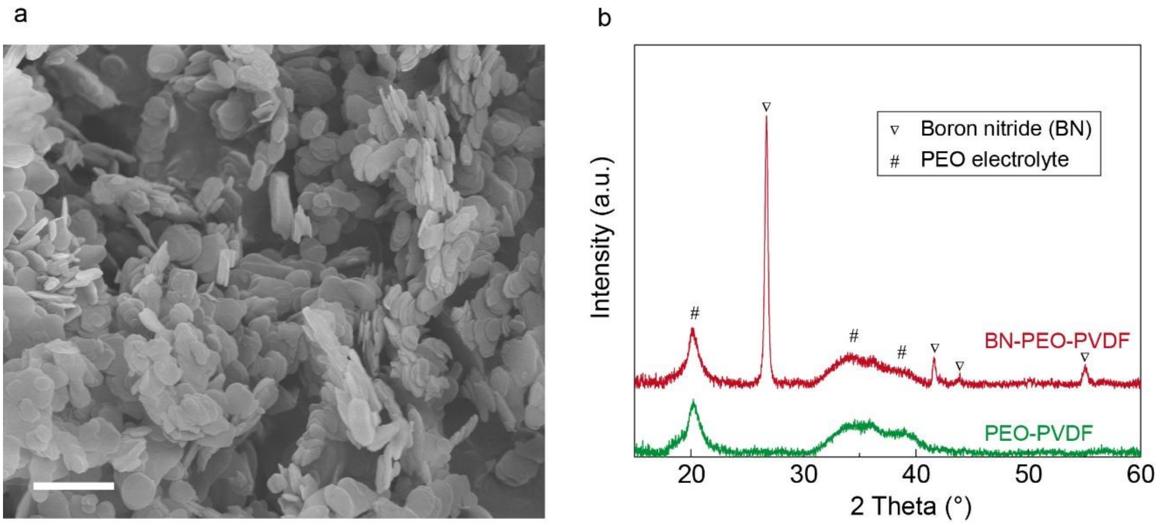

Figure S1. (a) SEM image of BN nanoflakes and (b) XRD patterns of PEO-PVDF and BN-PEO-PVDF electrolytes. Scale bar: 1 μm in (a)

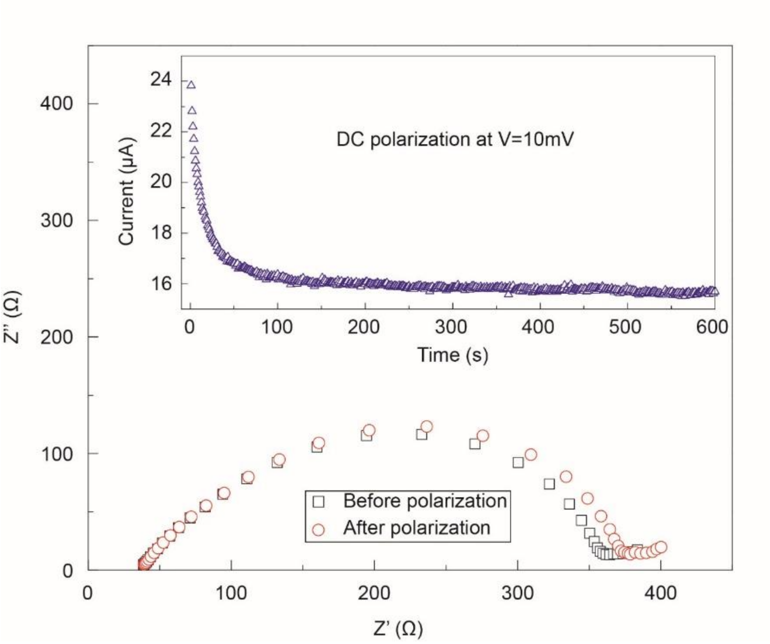

Figure S2. EIS plots of the electrolyte before and after DC polarization (an example for PEO-based electrolyte). The inset shows the current change with respect to time during DC polarization.

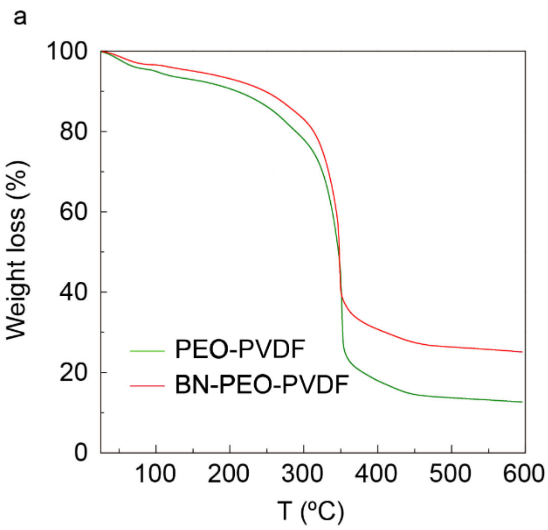

Figure S3. (a) TGA curves of PEO-PVDF and BN-PEO-PVDF electrolytes and (b) anodic stabilities of PEO-PVDF and BN-PEO-PVDF electrolytes (evaluated in Li-Al cells at 70 °C).

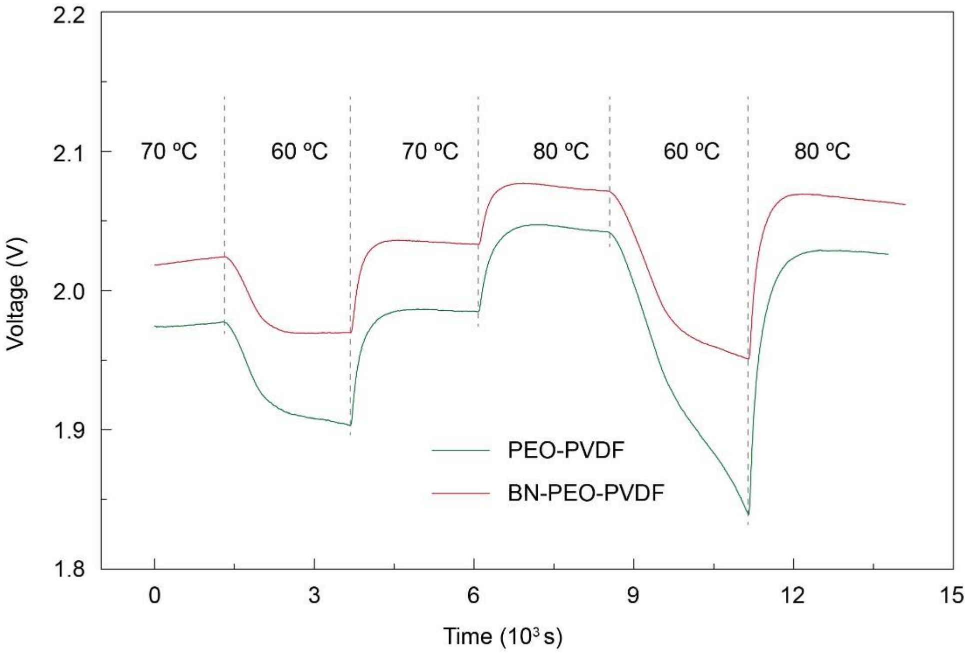

Figure S4. Changes of discharge curves (the lower potential plateau ~2.0 V) of Li-S cells with PEO-PVDF and BN-PEO-PVDF electrolytes with respect to temperature variations.

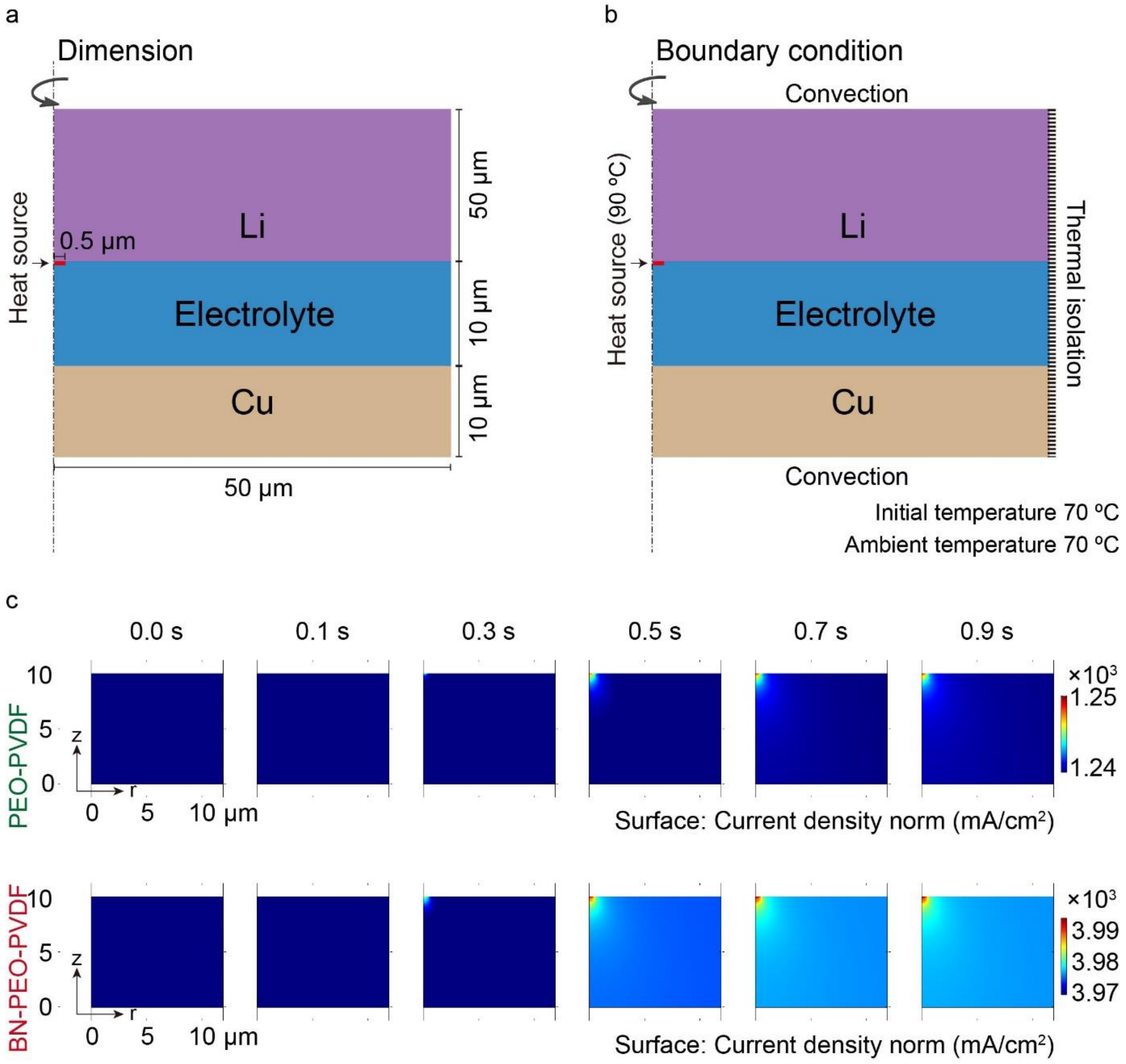

Figure S5. Modelling conditions for the current density changes upon a heat source formation in PEO-PVDF and BN-PEO-PVDF electrolytes: (a) dimension and (b) boundary conditions, (c) Dynamic current density distributions in the PEO-PVDF and BN-PEO-PVDF electrolytes with respect to time. The images of PEO-PVDF and BN-PEO-PVDF at 0.5 s are presented in Figure 3e in the manuscript. A supporting film is also provided as Video S1.

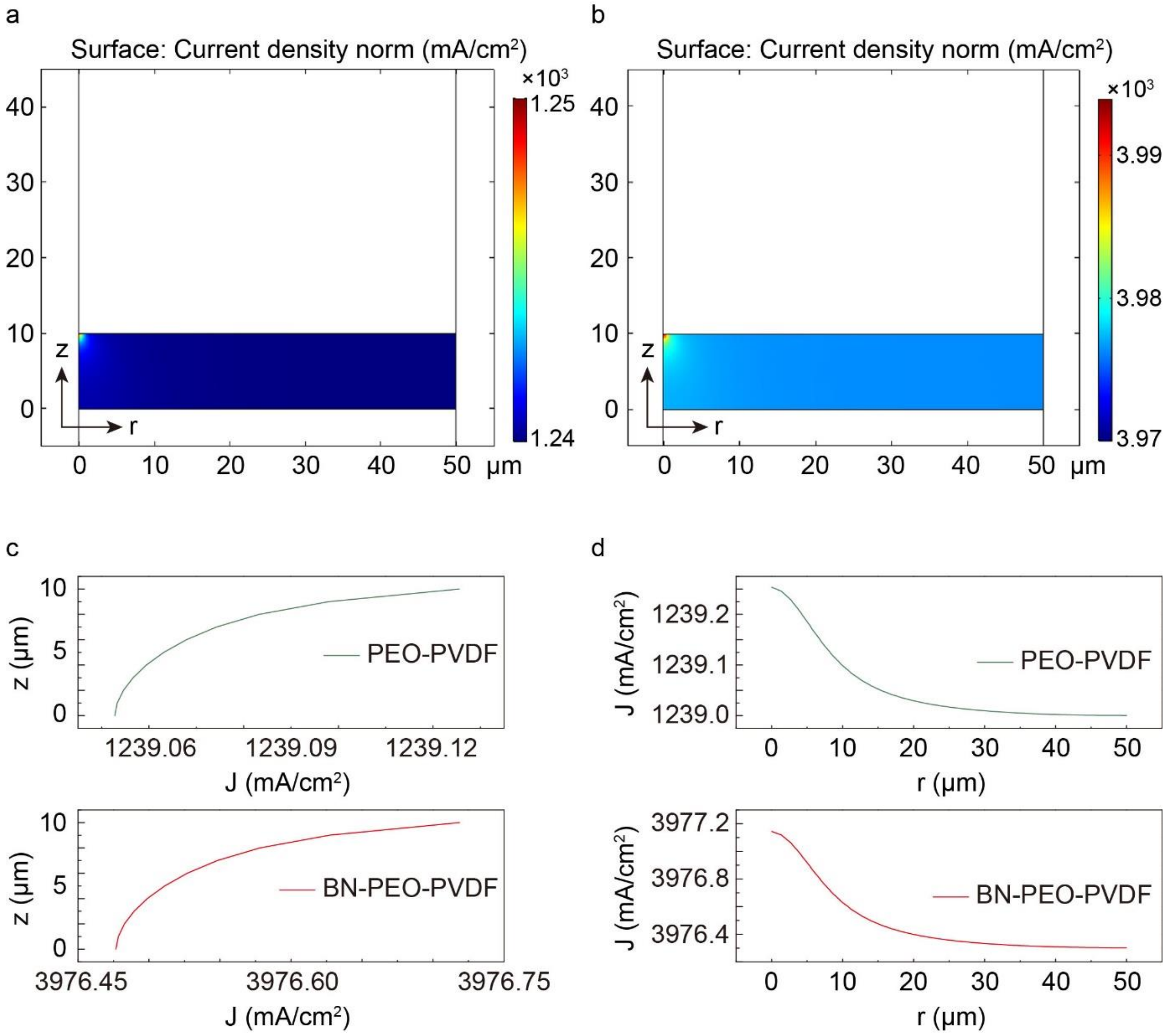

Figure S6. Current density (J) distributions in the PEO-PVDF (a) and BN-PEO-PVDF (b) electrolytes at the equilibrium state, corresponding J profiles along directions *z* (c) and *r* (d) in the PEO-PVDF and BN-PEO-PVDF electrolyte. In the manuscript (Figure 3e), J plots in (c) are normalized to the value of PEO-PVDF sample at z=0 μm and the J plots in (d) are normalized to the value of PEO-PVDF and BN-PEO-PVDF samples at r=50 μm for discussion.

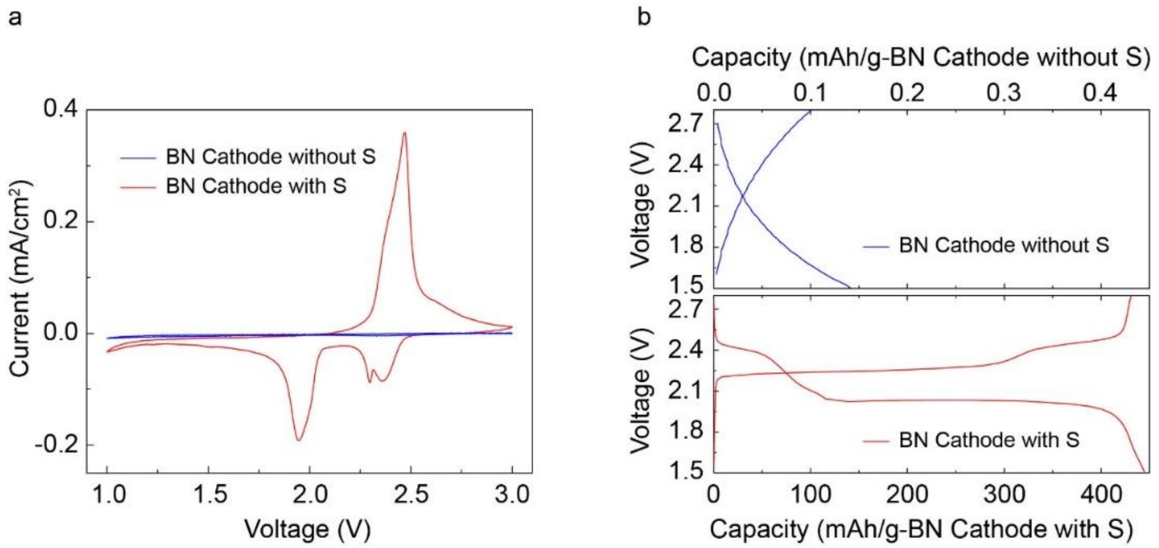

Figure S7. (a) CV curves and (b) charge-discharge profiles of solid-state BN-PEO-PVDF based cells with and without S in the cathode at 70 ^{o}C.

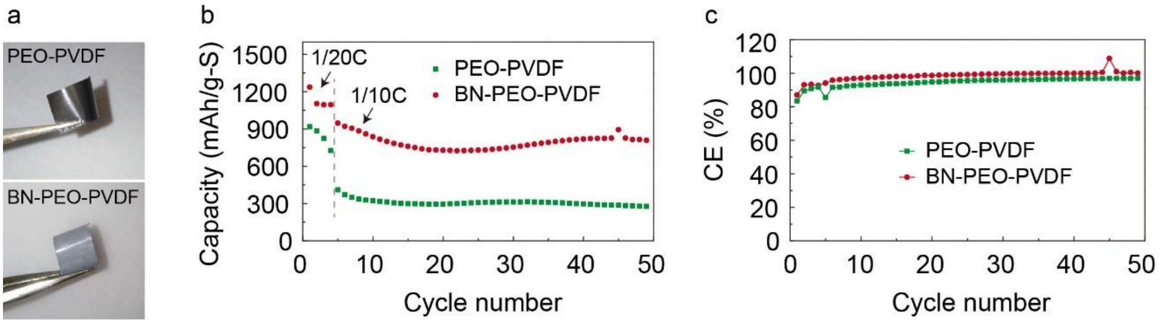

Figure S8. (a) Optical images of cathodes with PEO-PVDF and BN-PEO-PVDF electrolyte membranes, (b) Charge capacity decay and (c) coulombic efficiency (CE) change upon cycling for Li-S cells with PEO-PVDF and BN-PEO-PVDF electrolytes at 70 ^{o}C.

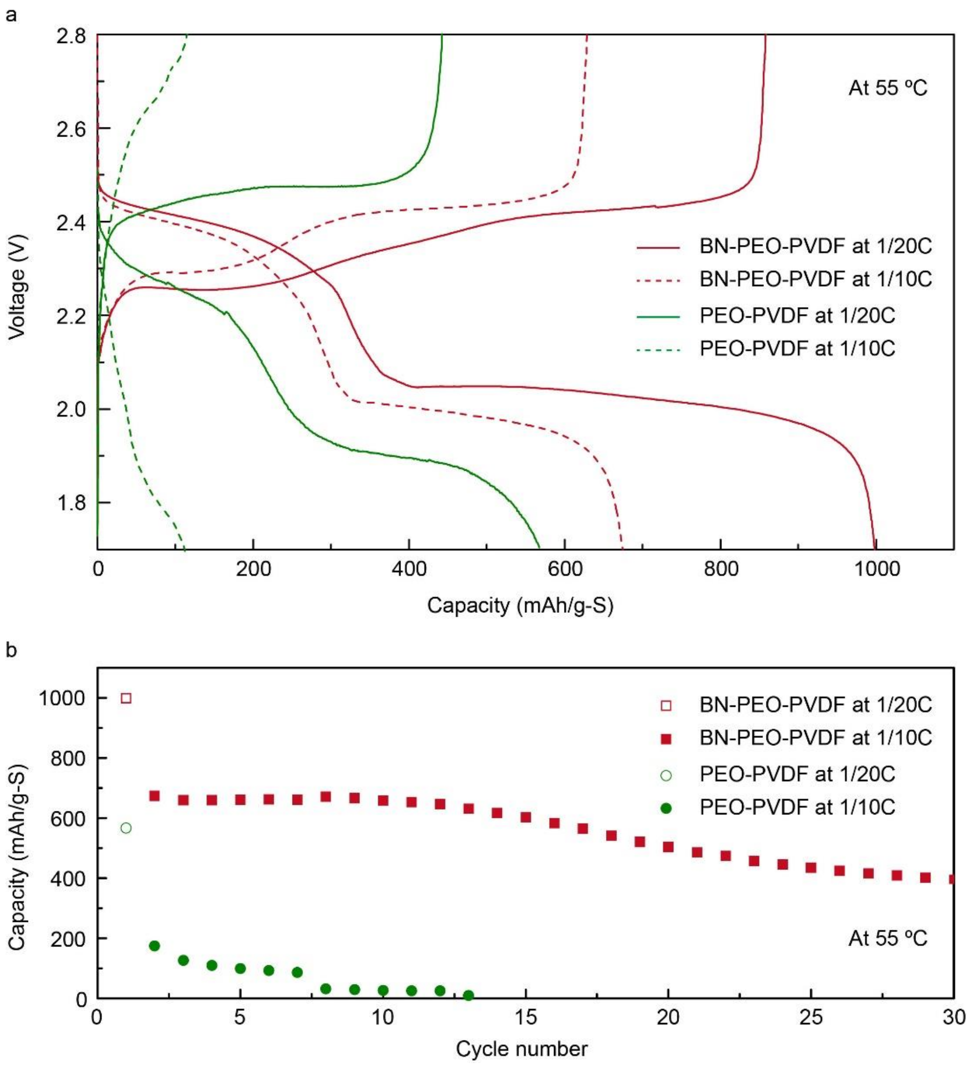

Figure S9. (a) Charge-discharge profiles and (b) capacity decay upon cycling for Li-S cells with PEO-PVDF and BN-PEO-PVDF electrolytes at 55 °C.

Table S1. Calculation of ion transfer numbers of BN-PEO-PVDF and PEO-PVDF electrolytes based on parameters obtained from EIS and DC polarization measurements. More details about the theory and calculation method can be found in literature.[1, 2]

Sample	Io/μA	Is/μA	Rbo/Ω	Rbs/Ω	R1o/Ω	R1s/Ω	V/mV	T_{Li}
BN-PEO-PVDF	17.0	12.6	39	39	501	510	10	**0.31**
PEO-PVDF	23.9	15.7	39	39	325	338	10	**0.31**

Table S2. Electrical conductivity (σ_e), thermal diffusivity (α), heat capacity (c) and thermal conductivity (κ) of BN, PEO-PVDF and BN-PEO-PVDF samples.

Sample	σ_e (10^{-10} Scm^{-1})	α (mm^{2}s^{-1})	c (Jg^{-1}K^{-1})	κ (Wm^{-1}K^{-1})
BN	(2.5 ±0.8)×10^{-2}	22.68 ±1.134[3]	0.39 ±0.019[3]	14.30 ±0.715[3]
PEO-PVDF	14.0 ±1.6	0.09 ±0.005	1.85 ±0.093	0.22 ±0.011
BN-PEO-PVDF	9.4 ±1.0	0.12 ±0.006	1.84 ±0.092	0.33 ±0.017

Supporting References:

[1] J. Evans, C. A. Vincent, P. G. Bruce, *Polymer* **1987**, 28, 2324.
[2] X. Judez, H. Zhang, C. Li, J. A. Gonzalez-Marcos, Z. Zhou, M. Armand, L. M. Rodriguez-Martinez, *J. Phys. Chem. Lett.* **2017**, 8, 1956.
[3] S. Mateti, K. Yang, X. Liu, S. Huang, J. Wang, L. H. Li, P. Hodgson, M. Zhou, J. He, Y. Chen, *Adv. Funct. Mater.* **2018**, 28, 1707556.
